# Idiopathic Hypoparathyroidism With Papillary Thyroid Carcinoma in a Young Male: A Rare Case Report

**DOI:** 10.3389/fendo.2020.569308

**Published:** 2020-12-15

**Authors:** Wenjie Chen, Liyun Chen, Tao Wei, Zhihui Li, Jianyong Lei, Jingqiang Zhu

**Affiliations:** ^1^ Thyroid and Parathyroid Surgery Center, West China Hospital of Sichuan University, Chengdu, China; ^2^ Outpatient Department, West China Hospital of Sichuan University, Chengdu, China

**Keywords:** idiopathic hypoparathyroidism, papillary thyroid carcinoma, parathyroid protection, central lymph node dissection, radioiodine ablation therapy

## Abstract

**Background:**

Idiopathic hypoparathyroidism (IHP) is a rare disorder that is diagnosed by excluding other possible etiologies. Thyroid surgery causes approximately 14–60% of all cases of hypoparathyroidism; of these, surgery for papillary thyroid carcinoma (PTC) is the most common reason. Here, we report an extremely rare case of IHP combined with PTC.

**Case presentation:**

A 22-year-old man presented with a history of uncontrollable extremity and facial numbness, spasm and twitch lasting for nine years. He had been misdiagnosed with epilepsy and gained no relief from antiepileptic therapy. The laboratory evaluation revealed reduced parathyroid hormone and serum calcium and elevated inorganic phosphorus. After considering IHP, ultrasound detected a solid hypoechoic and irregularly shaped nodule 13×8×9 mm in size in the upper pole of the right thyroid gland, and fine-needle aspiration biopsy indicated PTC. Then, the patient underwent surgical treatment and radioactive iodine ablation. The long-term treatment strategy consisted of oral levothyroxine for thyroid-stimulating hormone inhibition and oral calcium and vitamin D supplements for hypocalcemia control.

**Conclusion:**

We report a rare case of IHP combined with PTC in a 22-year-old male. Some experiences and lessons from our treatment procedure merit discussion, and we hope that our report can serve as a reference for the diagnosis and treatment of similar patients in the future.

## Background

Hypoparathyroidism is an uncommon endocrine disorder characterized by the absence or biological inactivity of parathyroid hormone (PTH), followed by hypocalcemia, hyperphosphatasemia and increased urinary calcium excretion (1, 2). The clinical presentation of hypoparathyroidism varies from mild paresthesia (burning, numbness or tingling sensation), carpopedal cramps, and epilepsy-like seizures to even life-threatening symptoms, such as laryngospasm and heart failure (1, 3). Idiopathic hypoparathyroidism (IHP) is rare with a reported prevalence of approximately 0.55–4.5 per 100,000 individuals in previous studies (4–6). Given that IHP is diagnosed by excluding other possible etiologies (7) and that the early symptoms are usually nonspecific and highly variable, misdiagnosis occurs occasionally in IHP. Following the occurrence of misdiagnosis, long-term uncontrolled symptoms may aggravate the condition and deteriorate the quality of life of the patients (8).

Thyroid carcinoma, especially papillary thyroid carcinoma (PTC), is the most common endocrine malignancy, and its incidence has dramatically increased in recent decades (9). While surgery is the preferred treatment for PTC, because the parathyroid glands are anatomically close to the thyroid gland and most of them are supplied by the upper or lower thyroid arteries, thyroidectomy and central lymph node (LN) dissection (CND) cause approximately 14–60% of all cases of hypoparathyroidism (10–12). Therefore, if a PTC patient has concurrent IHP, treating PTC while ensuring parathyroid function is a challenge worthy of consideration. To the best of our knowledge, there has been no case reports of IHP combined with thyroid carcinoma. Here, we report an extremely rare case of a patient with IHP combined with PTC in mainland China who was initially misdiagnosed with epilepsy. After the diagnosis, we performed surgery and radioiodine ablation (RAI) for PTC in this patient, and we hope that our treatment procedures and initial outcomes can serve as a reference for the diagnosis and treatment of similar patients.

## Case Presentation

In February 2019, a 22-year-old man presented to our emergency department with uncontrollable extremity and facial numbness, spasm and twitching lasting for half an hour. He had a history of the above-mentioned symptoms for 9 years and aggravation for 1 year (1–2 times a day, no consciousness disorder). Previously, multiple electroencephalogram examinations were performed at his local hospital, and the results showed occasional short-term sharp waves in his forehead. In the absence of laboratory tests of parathyroid hormone (PTH) and serum calcium, he was empirically diagnosed with epilepsy and treated with oral sodium valproate (1,000 mg qd) without improvement; hence, he decided to stop taking the medicine 6 months prior.

After visiting our hospital, the laboratory examination upon admission revealed the following: PTH 1.34 pmol/L (reference range, 1.60–6.90 pmol/L), serum calcium 1.55 mmol/L (reference range, 2.10–2.70 mmol/L), inorganic phosphorus (IPOS) 1.84 mmol/L (reference range, 0.81–1.45 mmol/L), magnesium 0.84 mmol/L (reference range, 0.67–1.04 mmol/L) and 25-hydroxyvitamin D3 63.11 nmol/L (reference range, 47.70–144.00 pmol/L). The urinary system ultrasonography was negative. He had no history of neck surgery, neck radiotherapy, parathyroid disease or hereditary disease. In addition, the patient had no special facial features, no history of macrovascular abnormalities, no history of serious fungal infection, such as *Candida* and *Pneumocystis carinii*, and an absolute count of peripheral blood lymphocytes in the normal range; thus, the possibility of congenital hypo-parathyroidism, such as De George’s syndrome, was excluded. Furthermore, no abnormal family history or autoimmune history was presented. Given the patient’s obvious extremity and facial numbness and spasm and because the laboratory tests showed hypoparathyroidism, hypocalcemia and hyperphosphatasemia, after excluding other possible etiologies, a diagnosis of IHP was suspected, and his symptoms were alleviated by urgent intravenous calcium gluconate (20 mg IV).

**Figure 1:**
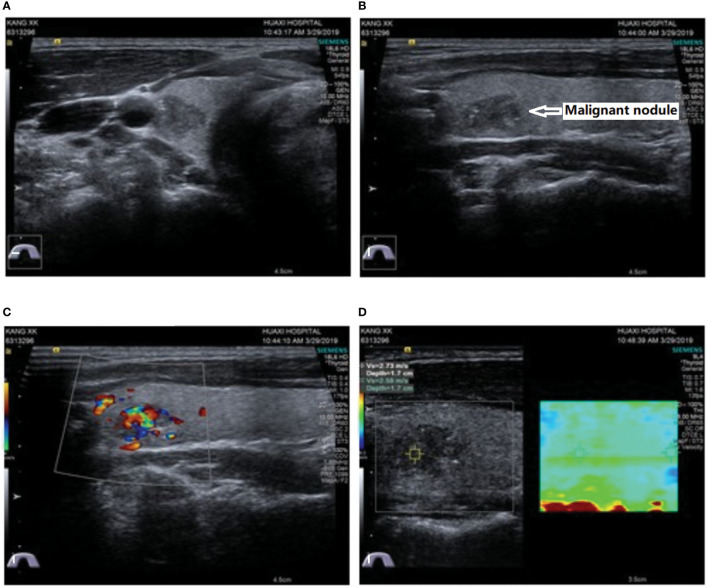
Ultrasound showed a solid hypoechoic and irregularly shaped nodule 13 × 8 × 9 mm in size with microcalcifications, a taller-than-wide shape and suspicious extracapsular extension located in the upper pole of the right thyroid gland. **(A)** Transverse view; **(B)** longitudinal view; **(C)** Doppler blood flow signals; **(D)** elastography.

To comprehensively evaluate the condition of IHP, cervical ultrasonography was performed and showed negative results for the parathyroid glands, but a solid hypoechoic mass measuring 13×8×9 mm with irregular margins, macrocalcifications, a taller than wide shape and suspicious extracapsular extension was accidentally found in the upper pole of the right thyroid gland (**Figure 1**). Then, fine-needle aspiration (FNA) was performed on this nodule, and PTC was suspected according to the cytology results. In addition, all thyroid function test values were within the normal reference range, as well as the levels of thyroid peroxidase antibody (TPOAb) and anti-thyroglobulin antibody (TgAb). Subsequently, a therapeutic measure of oral Caltrate D 600 mg bid and oral calcitriol 0.25 µg qd was administered until the preoperative time to control symptomatic hypocalcemia.

Multidisciplinary discussions focused on how to address PTC and protect the parathyroid function in the presence of IHP. Given that the size of the primary lesion was greater than 1 cm, active surveillance and thermal ablation were ruled out, and surgical intervention was finally considered. According to the 2015^th^ ATA guidelines, for patients with PTC >1 cm and <4 cm without high invasive characteristics, the initial surgical procedure can be either total thyroidectomy or lobectomy. To reduce the risk of postoperative hypoparathyroidism, hemithyroidectomy was determined as a basic surgical procedure. However, the primary premise is a sufficient surgical resection range to avoid the recurrence of the tumor and a secondary operation, which may also increase the risk of parathyroid injury. Therefore, the final resection range should be comprehensively judged by the preoperative and intraoperative findings. Besides, the protection of parathyroid gland during operation lies in careful anatomy and avoidance of parathyroid and supplied vascular injury.

As mentioned above, sufficient preoperative evaluations, including neck ultrasonography, thyroid function tests and FNA, were performed, and normal bilateral vocal cord activity was confirmed by preoperative vocal cord laryngoscopy. In addition, the serum calcium and PTH levels were evaluated again, and the PTH value was still at an abnormally low level (1.26 pmol/L), while the serum calcium value returned to within the normal range (2.24 mmol/L) after the calcium and calcitriol supplementation.

**Figure 2:**
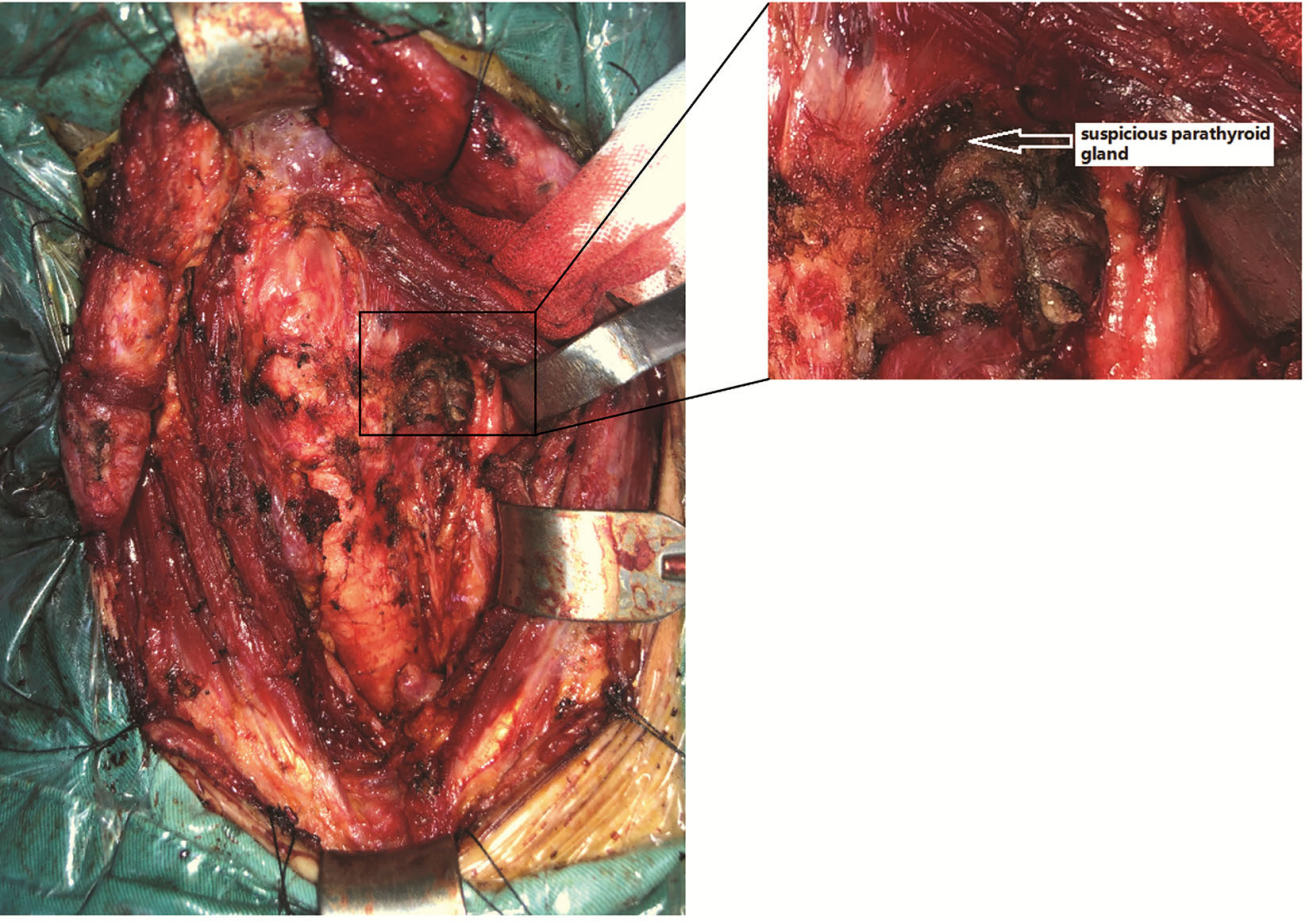
A suspicious parathyroid gland was found in the superior left area of the thyroid bed.

During the operation, first, right hemithyroidectomy and isthmusectomy were performed. After dissecting the isolated thyroid gland evenly according to a thickness of 1-2 mm, a hard, white nodule measuring approximately 13×10×10 mm in size was found in the upper pole of right thyroid gland. Moreover, several fused LNs ranging in size from 2 to 12 mm were found in the right central and pretracheal areas with a maximum diameter of 12×10×8 mm. Then, the tissues in the right central and pretracheal areas were examined by intraoperative cryosectioning, and a high LN metastasis ratio was found (5/5 and 6/8, respectively). Considering the possibility of LN metastasis in the contralateral central area, the patient could have needed RAI ablation after the operation; thus, total thyroidectomy plus bilateral CND were finally performed. After the excision of the tissue, a careful search in the dissected thyroid (slice thickness 1–2 mm) and central tissue was routinely performed to identify mistakenly cut parathyroid glands. During the operation, only one suspicious parathyroid was found in the superior left position (**Figure 2**), which was a small brown yellow nodule with a soft texture and approximately 3×2×2 mm in size that was supplied by the posterior branch of the superior thyroid artery; this gland was kept in situ. No suspicious parathyroid glands were found in the dissected thyroid or central tissue. Considering the diagnosis of IHP, we speculate that this patient only has one parathyroid gland, while the other parathyroid glands may be absent due to congenital or acquired factors. Intraoperative nerve monitoring (Medtronic, NIM-Response 3.0) was recommended to prognosticate recurrent laryngeal nerve (RLN) function.

The postoperative paraffin section examination showed a hard, grayish-white nodule measuring 13 mm in diameter with an extracapsular extension in the right thyroid gland, and the histological findings showed that the tumor cells were arranged in branched papillary structures, and the nucleus was ground glass-like with pseudoinclusion bodies and nuclear grooves; granular calcification was scattered between the tumor cells. Furthermore, a high metastatic ratio was found in the central LNs (5/5 in the right central LNs, 6/8 in the pretracheal LNs and 2/3 in the left central LNs), which ranged in diameter from 2–12 mm. A protocol of oral levothyroxine 100 µg qd was applied for long-term TSH inhibition. The postoperative calcium supplementation strategy consisted of calcium gluconate 20 mg IV bid and oral calcitriol 0.25 µg bid until discharge. Subsequently, the protocol was changed back to oral Caltrate D 600 mg bid and oral calcitriol 0.25 µg qd for the long term.

**Figure 3:**
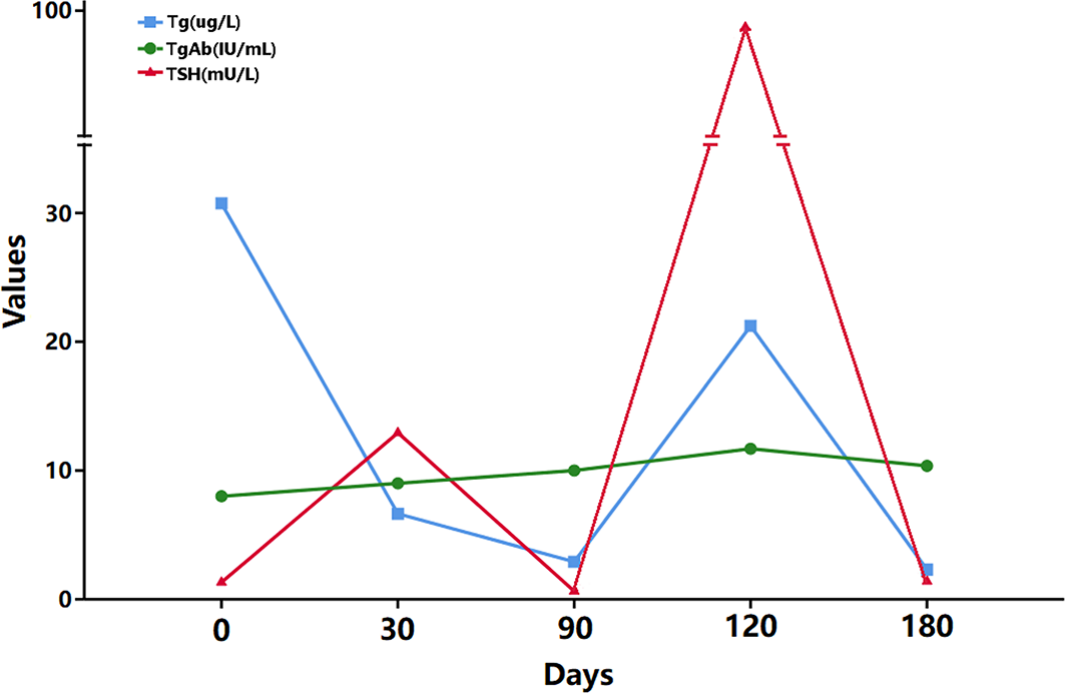
TSH, hTg, and TgAb levels. The patient received RAI therapy 120 days after the operation.

Three months after the operation, due to the high metastatic ratio of LNs, the thyroid function tests indicated stimulated TSH > 100 mU/L and stimulated human thyroglobulin (hTg) 21.23 µg/L, and ^131^ whole-body diagnostic imaging with a dose of 5 mCi revealed no abnormal radiation uptake anywhere in the body, except for the thyroid bed area; thus, RAI therapy was considered. Subsequently, RAI therapy with 150 mCi was performed. The patient was discharged 4 days after the treatment, and despite the long-term follow-up, the hTg value remained outside of the desired range recommended by the 2015^th^ ATA guidelines (**Figure 3**).

**Figure 4:**
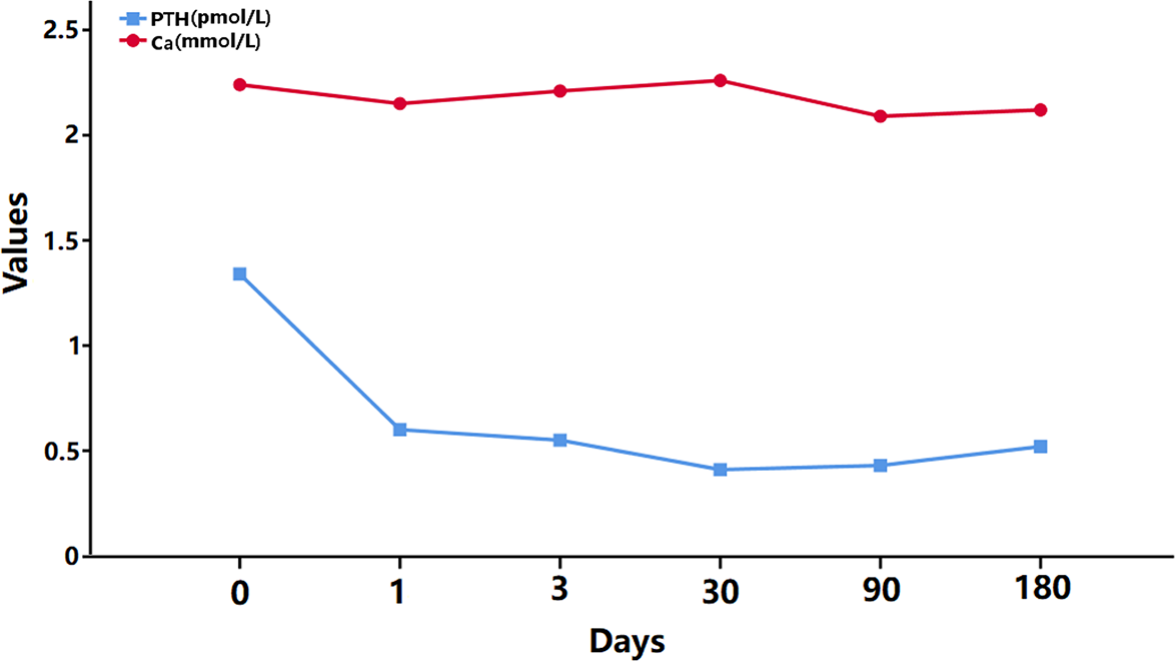
PTH and Ca levels.

During the follow-up period, the serum calcium and PTH values were continuously monitored, and the variation curve is shown in **Figure 4**. The postoperative PTH value was lower than the preoperative value, while the calcium level remained normal because of the routine oral calcium and vitamin D supplementation. Moreover, significant symptoms of calcium deficiency were no longer mentioned by the patient, and no other surgery-related complications were found.

## Discussion

To the best of our knowledge, this is the first report of a patient with IHP combined with PTC, and we describe the treatment procedure and initial outcomes in great detail. In this rare case, the patient was initially empirically misdiagnosed with epilepsy at a local medical unit and received antiepileptic treatment for up to nine years. After the diagnosis of IHP and PTC, the patient underwent surgery and RAI for PTC, followed by long-term TSH inhibition therapy and oral calcium and vitamin D supplementation.

This case underscores several critical points that should be considered. First, hypoparathyroidism and hypocalcemia should be considered in patients with unexplained convulsive symptoms, and routine examinations of calcium, phosphorus and PTH are economical and effective for diagnosis and differential diagnosis, especially when other treatments fail to achieve the desired goals. In addition, extensive central nervous system calcification is common in patients with hypoparathyroidism; these lesions can be detected by CT, and prevalence rates of 52–74% have been previously reported (13, 14). IHP is a rare disease with various clinical manifestations, which may predispose patients to misdiagnosis if clinicians fail to consider this disease (6). Hence, a regular review of IHP is necessary for clinicians, especially inexperienced grassroots doctors, emergency physicians and neurologists.

Second, imaging evaluations should not be ignored when evaluating functional organs. Generally, the diagnosis of hypoparathyroidism depends on low PTH levels in the context of hypocalcemia, and imaging evaluations are not routinely suggested in the guidelines (15, 16). However, in the present study, because of the “extra” neck ultrasonography examination, we found the thyroid focus, although this focus was not on the parathyroid. Similar to thyroid function tests and ultrasound, which are routinely combined to assess thyroid disorders, we suggest that laboratory tests and imaging examinations should be combined to evaluate functional organs for disease systematically and comprehensively.

Then, choosing suitable treatment measures for this patient in the present study was a focus of heated discussion. Because of the presence of IHP in this patient, active surveillance, thermal ablation and surgical treatment were all considered. According to the 2015^th^ ATA guidelines, active surveillance is suggested only for select low-risk papillary thyroid microcarcinoma (PTMC) patients (17–19); thus, active surveillance was not considered for this patient. In addition, evidence is still insufficient to suggest that thermal ablation is effective for primary PTC, which is also not recommended as a routinely preferred measure for PTC in the Chinese and Korean guidelines (20, 21); thus, we also ruled out thermal ablation. However, the question of whether the guidelines and expert consensus are still applicable in special cases, such as patients with IHP combined with PTC, remains unanswered. Because there were no other reports from which we could learn, surgery was our final choice. As the ultrasonography shown that the diameter of the tumor was 13×8×9mm, hemithyroidectomy plus isthmusectomy was selected as the initial operation (17). However, total thyroidectomy plus bilateral CND were finally performed according to the intraoperative findings. Previous studies at our center have found that pretracheal LN metastasisisa predictor of contralateral central LN metastasis, especially in patients with several pretracheal metastatic LNs≥3 (22, 23). In addition, the possibility of RAI therapy requires total thyroidectomy. The surgical details are described above.

The fourth important question is as follows: Does this patient need to be treated with RAI? Does RAI therapy affect parathyroid function? Generally, RAI is considered for patients with the pathological stage of pT1bN1aM0 according to the 2015^th^ ATA guidelines (17), and this consideration could be more favored for patients with more than 5 metastatic nodes or metastatic nodes >1 cm in diameter (17, 24). In our case, in total, 13 metastatic LNs were found in the central area, and the metastatic LNs were larger than 10 mm in diameter; thus, RAI therapy was considered an option. In addition, the side effects of RAI, such as sialadenitis, xerostomia and nausea, are well known (25, 26), but the effects on parathyroid function remain controversial. Several studies have shown hypo- or hyperparathyroidism following RAI treatment, but most findings were presented in the form of case reports (27–30). Mortensen LS’s study investigated parathyroid function over the long term (8–12 years) after RAI for benign thyroid diseases, and no significant changes in calcium or PTH were found (31). Szumowski P’s study (32) found a transient decline in PTH without a significant change in calcium, phosphates and symptoms following high-dose (100 mCi or 150 mCi) RAI therapy for toxic and nontoxic goiters. Similar results were reported in Guven A’s studies focusing on thyroid cancer (33). Altogether, we considered RAI to have no significant effect on the parathyroid glands; thus, RAI therapy with a dose of 150 mCi was performed, and while there was no significant change in the iPTH value at 3 months after RAI therapy, long-term follow-up of the parathyroid function was still needed.

The final important finding is associated with the postoperative hTg level. As shown in **Figure 2**, the value of hTg was stable at a relatively high level after the total thyroidectomy plus bilateral CND and did not fall within the ideal range (<1 ng/mL) after RAI therapy. Combined with the preoperative ultrasound findings and postoperative pathological results of this patient, the final important issue was considered to be awareness of the occurrence of lateral cervical LN metastasis during the follow-up period. Previous studies have shown that the metastasis of lateral cervical LNs in PTC is associated with the tumor size, upper pole tumors, T4 stage, pathological central LN metastasis, extrathyroidal extension, etc (34–39).. Of these factors, central LN metastasis and upper pole tumors have shown relatively high odds ratios for the prediction of lateral LN metastasis in most studies. Moreover, an LN metastasis ratio >0.5 and several positive central LNs ≥2 were also highly predictive of lateral LN metastasis (38, 40). In the present study, the primary focus was on extracapsular extension, which was located in the upper pole of the right thyroid gland, and a high metastatic ratio was found in the central LNs (13/16 in total, 81.3%). Hence, lateral LN metastasis was highly suspected in this patient, and close follow-up is in progress.

## Conclusion

IHP combined with PTC is an extremely rare disorder. We are the first to report the treatment procedures and initial outcomes in a young patient with IHP combined with PTC. Some experiences and lessons from our therapeutic strategies merit discussion, and we hope that our report can serve as a reference for the diagnosis and treatment of similar patients in the future.

## Data Availability Statement

The original contributions presented in the study are included in the article/supplementary materials; further inquiries can be directed to the corresponding author.

## Ethics Statement

The studies involving human participants were reviewed and approved by the Ethics Committee of West China Hospital of Sichuan University. The patients/participants provided their written informed consent to participate in this study. No animal studies are presented in this manuscript. No potentially identifiable human images or data are presented in this study.

## Author Contributions

All authors substantially contributed to the conception and design, acquisition of the data, or analysis and interpretation of the data, participated in drafting or critically revising the article for important intellectual content, and approved the final version for publication. All authors agree to be accountable for the content of the work. All authors contributed to the article and approved the submitted version.

## Funding

This study was supported by grants from the National Natural Science Foundation (81702646), Post-Doctor Research Project, West China Hospital, Sichuan University (2019HXBH043), and Sichuan Province Science and Technology Project of China (2020YFS0208).

## Conflict of Interest

The authors declare that the research was conducted in the absence of any commercial or financial relationships that could be construed as potential conflicts of interest.

## Abbreviations

IHP, idiopathic hypoparathyroidism; PTC, papillary thyroid carcinoma; PTH, parathyroid hormone; LN, lymph node; CND, central lymph node dissection; RAI, radioiodine ablation; IPOS, inorganic phosphorus; FNA, fine-needle aspiration; TPOAb, thyroid peroxidase antibody; TgAb, anti-thyroglobulin antibody; PTMC, papillary thyroid microcarcinoma.
